# Crystal structure of (*Z*)-2,3-di­chloro-1,4-bis­(4-meth­oxy­phen­yl)but-2-ene-1,4-dione

**DOI:** 10.1107/S1600536814018790

**Published:** 2014-08-23

**Authors:** Ram K. Tittal, Satish Kumar, R. N. Ram

**Affiliations:** a Department of Chemistry, Indian Institute of Technology Delhi, Hauzkhas, New Delhi 110 016, India; b Department of Chemistry, St Stephen’s College, University Enclave, Delhi 110 007, India

**Keywords:** crystal structure, 2,3-di­chloro­but-2-ene-1,4-dione, di­chloro­methyl radical, CuCl/bpy, tri­chloro­methyl groups, stereoselectivity

## Abstract

The title compound, C_18_H_14_Cl_2_O_4_, adopts a *Z* conformation around the cental C=C bond. The two aromatic rings of the mol­ecule are nearly perpendicular to each other, with a dihedral angle between of 86.22 (14)°. The meth­oxy substituents lie close to the plane of the attached benzene rings. The C(ar)—C(ar)—O—C(Me) torsion angles are −2.4 (7) and 7.5 (6)°. Weak C—H⋯O inter­actions link the mol­ecules forming a three-dimensional network. The crystal packing also displays short [3.160 (3) Å] Cl⋯O halogen-bonding contacts between mol­ecules related by the screw axis. The structure exhibits disorder of one carbonyl O atom with a refined occupancy ratio of 0.21 (6):0.79 (6).

## Related literature   

For a review of radical reactions, see: Clark (2002 ▶); Ram & Tittal (2014*a* ▶,*b* ▶); Pintauer & Matyjaszewski (2008 ▶). For details of the synthesis, see: Kurosawa & Yamaguchi (1981 ▶); Ram *et al.* (2007 ▶). For halogen-bond inter­actions, see: Agarwal *et al.* (2014 ▶). For a similar structure and short aromatic contacts, see: Tittal *et al.* (2014 ▶).
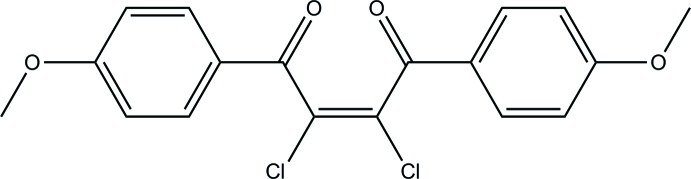


## Experimental   

### Crystal data  

C_18_H_14_Cl_2_O_4_
*M* *_r_* = 365.19
Monoclinic, 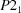

*a* = 8.877 (3) Å
*b* = 9.914 (3) Å
*c* = 10.294 (3) Å
β = 110.565 (5)°
*V* = 848.3 (5) Å^3^
*Z* = 2
Mo *K*α radiation
μ = 0.40 mm^−1^
*T* = 273 K
0.31 × 0.23 × 0.14 mm

### Data collection  

Bruker SMART APEX CCD diffractometer
Absorption correction: multi-scan (*SADABS*; Bruker, 2000 ▶) *T*_min_ = 0.860, *T*_max_ = 1.000
9879 measured reflections
4012 independent reflections
3755 reflections with *I* > 2σ(*I*)
*R*_int_ = 0.025

### Refinement  

*R*[*F* ^2^ > 2σ(*F* ^2^)] = 0.057
*wR*(*F* ^2^) = 0.134
*S* = 1.17
4012 reflections
223 parameters
2 restraints
H-atom parameters constrained
Δρ_max_ = 0.41 e Å^−3^
Δρ_min_ = −0.17 e Å^−3^
Absolute structure: Flack *x* determined using 1548 quotients [(*I* ^+^)−(*I* ^−^)]/[(*I* ^+^)+(*I* ^−^)] (Parsons *et al.*, 2013 ▶)
Absolute structure parameter: 0.01 (2)

### 

Data collection: *SMART* (Bruker, 2000 ▶); cell refinement: *SAINT* (Bruker, 2000 ▶); data reduction: *SAINT*; program(s) used to solve structure: *SHELXS97* (Sheldrick, 2008 ▶); program(s) used to refine structure: *SHELXL97* (Sheldrick, 2008 ▶); molecular graphics: *OLEX2* (Dolomanov *et al.*, 2009 ▶) and *Mercury* (Macrae *et al.*, 2008 ▶); software used to prepare material for publication: *OLEX2*, *PLATON* (Spek, 2009 ▶) and *publCIF* (Westrip, 2010 ▶).

## Supplementary Material

Crystal structure: contains datablock(s) I. DOI: 10.1107/S1600536814018790/fy2117sup1.cif

Structure factors: contains datablock(s) I. DOI: 10.1107/S1600536814018790/fy2117Isup2.hkl

Supporting information file. DOI: 10.1107/S1600536814018790/fy2117Isup3.cml

**Fig. 1.**
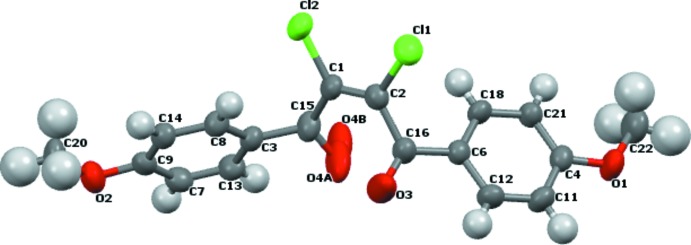
Molecular structure of the title compound, with atom labels and 50% probability displacement ellipsoids for non-H atoms.

. DOI: 10.1107/S1600536814018790/fy2117fig1.tif
Mol­ecular structure of the title compound, with atom labels and 50% probability displacement ellipsoids for non-H atoms.

**Fig. 2.**
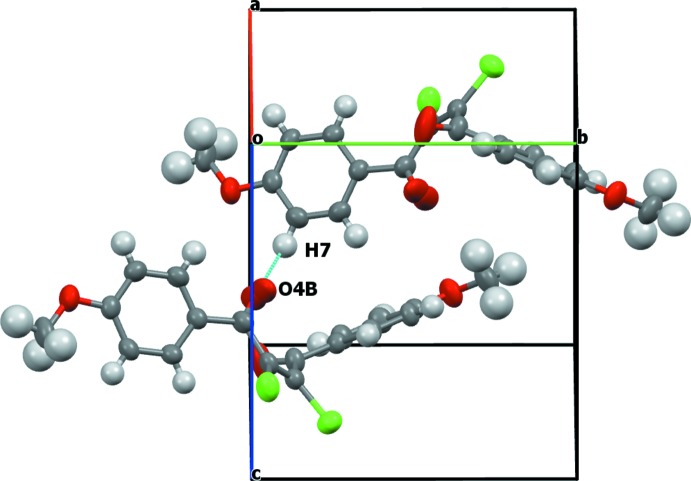
A view of the crystal packing of the title compound. A weak C—H···O hydrogen bond is shown as a dashed line.

. DOI: 10.1107/S1600536814018790/fy2117fig2.tif
A view of the crystal packing of the title compound. A weak C—H⋯O hydrogen bond is shown as a dashed line.

**Fig. 3.**
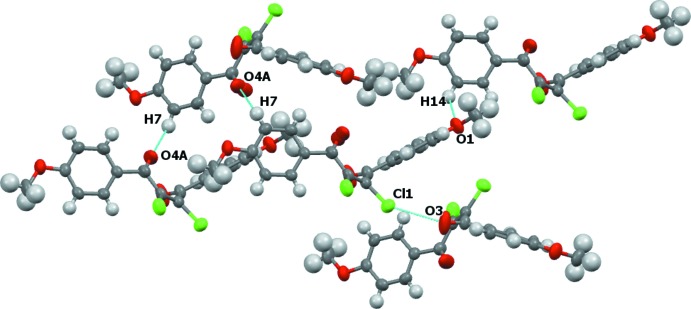
Part of the structure of the title compound showing O····Cl and C—H····O interactions.

. DOI: 10.1107/S1600536814018790/fy2117fig3.tif
Part of the structure of the title compound showing O⋯·Cl and C—H⋯·O inter­actions.

CCDC reference: 1019996

Additional supporting information:  crystallographic information; 3D view; checkCIF report

## Figures and Tables

**Table 1:** Hydrogen-bond geometry (Å, °)

*D*—H⋯*A*	*D*—H	H⋯*A*	*D*⋯*A*	*D*—H⋯*A*
C7—H7⋯O4*A* ^i^	0.93	2.49	3.23 (3)	137
C7—H7⋯O4*B* ^i^	0.93	2.62	3.245 (9)	125
C12—H12⋯O1^ii^	0.93	2.66	3.488 (5)	149
C14—H14⋯O1^iii^	0.93	2.72	3.363 (5)	127

Symmetry codes: (i) 
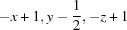
; (ii) 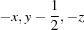; (iii) 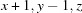.

## supplementary crystallographic information

### S1. Chemical context

Tri­chloro­methyl groups are known to generate free radicals through homolysis of a C—Cl bond with relative ease in presence of radical initiators or UV-light or redox active metal salts (such as CuCl) or its complexes. A number of research papers are available that show radical inter­mediates produced from tri­chloro­methyl group containing compounds. For example, CuCl or its complexes with bi­pyridine or bi- or tridendentate amine ligands have been used with tri­chloro­methyl groups on a variety of organic substrates under non reducing conditions to generate free radicals. Such radicals have been reported to undergo intra­molecular and inter­molecular cyclization or addition reactions along with mono- and/or-di redution products (Clark, 2002; Ram *et al.*,2007). The radicals produced in these reactions can also acts as a radical initiator in ATRP (Pintauer & Matyjaszewski, 2008). However, the presence of relatively better leaving group at the β-position of the radical centre leads to predominantly rearrangement and/or fragmentation reactions through the inter­mediate formation of contact ion pairs (Ram & Tittal, 2014*a*,*b*). It is important to mention here that 2,2,2-tri­chloro­ethyl­alkyl ethers and tri­chloro­methyl carbinols with no leaving group at β-position to the tri­chloro­methyl around carbon carbon double bond undergoes 1,2-H shift under similar conditions through the inter­mediate copper-carbenoid species (Ram & Tittal, 2014*b*). Keeping in view the above discussion, we have decided to study the behavior of the radicals produced from substituted tri­chloro­methyl compounds with no suitably located carbon-carbon double bond and leaving group or any hydrogen atom at the β-position of the radical centre in order to restrict the inter­molecular or intra­molecular addition; ATRP; rearrangement and/or fragmentation or 1,2-H shift. The major product obtained under such conditions is reported here.

### S2. Structural commentary

In the asymmetric unit (Fig. 1) the moleculecule adopts Z conformation about the C=C bond: C8═C9. The two aromatic rings are nearly perpendicular with an inter­planar angle between the two phenyl rings of 86.22 (14)°. The two meth­oxy substituents lie close to the plane of the attached benzene rings. The C(ar)—C(ar)—O—C(Me) torsion angles are -2.4 (7)° (C21—C4—O1—C22) and 7.5 (6)° (C14—C9—O2—C20). The structure exhibits disorder of carbonyl group with a refined occupancy ratio of 0.21 (6): 0.79 (6).

### S3. Supra­molecular features

The crystal packing is stablized by short halogen bond Cl···O inter­actions (Fig. 2) [3.160 (3)Å]. The crystal structure of also displays inter­molecular C(ar)—H···O (Table 1, Fig. 3) short contacts.

### S5. Synthesis and crystallization

A two-neck round bottom flask fitted with a rubber septum was charged with CuCl (0.8 g, 0.008mol), 2,2'-bi­pyridine (bpy, 1.25 g, 0.008 mol) under continuous flow of nitro­gen followed by addition of 1 mL dry di­chloro­ethane (DCE) or benzene to ensure CuCl-bpy complex formation. To this reaction flask a solution of the 2,2,2-tri­chloro-1-(4-meth­oxy-phenyl)-ethanone (0.004 mol) in dry DCE or benzene (5 mL) was injected through the septum with the help of a syringe and the reaction mixture was heated at reflux with stirring under a slow and continuous flow of nitro­gen. The completion of the reaction was indicated by TLC (1-2 h). The reaction mixture was cooled and filtered through a celite pad. The filtrate was evaporated under reduced pressure and purified on a silica gel column chromatography using *n*-hexane and ethyl­acetate mixture as the solvent to get title compound in 60 or 70% isolated yields in DCE or benzene, respectively. Suitable single crystals were obtained from a chloro­form:henxane (3:7) mixture by slow evaporation at room temperature. Melting point 132°C, literature melting point 132-134°C (Kurosawa & Yamaguchi, 1981).

### S6. Refinement

H atoms were placed in calculated positions and refined using riding model, with C—H 0.93 Å and U_iso_(H) = 1.2U_eq_(C) for aromatic C—H. Methyl groups were refined as idealised rotating groups, with C—H = 0.96 Å and U_iso_(H) = 1.5U_eq_(C). Disorder was modeled for O4 atom of the carbonyl group over two sets of sites with minor:major occupancy ratio of 0.21 (6): 0.79 (6). Similarity restraints were used for the C═O bond distances using SADI. Anisotropic displacement parameters of the minor component of oxygen atom O4 were constrained to those of the major component using EADP.

### Crystal data

**Table:**

C_18_H_14_Cl_2_O_4_	*F*(000) = 376
*M**_r_* = 365.19	*D*_x_ = 1.430 Mg m^−^^3^
Monoclinic, *P*2_1_	Mo *K*α radiation, λ = 0.71073 Å
*a* = 8.877 (3) Å	Cell parameters from 5754 reflections
*b* = 9.914 (3) Å	θ = 3.2–26.1°
*c* = 10.294 (3) Å	µ = 0.40 mm^−^^1^
β = 110.565 (5)°	*T* = 273 K
*V* = 848.3 (5) Å^3^	Block, colorless
*Z* = 2	0.31 × 0.23 × 0.14 mm

### Data collection

**Table:**

Bruker SMART APEX CCD diffractometer	3755 reflections with *I* > 2σ(*I*)
Radiation source: fine-focus sealed tube	*R*_int_ = 0.025
φ and ω scans	θ_max_ = 28.3°, θ_min_ = 2.1°
Absorption correction: multi-scan (*SADABS*; Bruker, 2000)	*h* = −11→11
*T*_min_ = 0.860, *T*_max_ = 1.000	*k* = −12→13
9879 measured reflections	*l* = −13→13
4012 independent reflections	

### Refinement

**Table:**

Refinement on *F*^2^	Hydrogen site location: inferred from neighbouring sites
Least-squares matrix: full	H-atom parameters constrained
*R*[*F*^2^ > 2σ(*F*^2^)] = 0.057	*w* = 1/[σ^2^(*F*_o_^2^) + (0.0741*P*)^2^ + 0.0555*P*] where *P* = (*F*_o_^2^ + 2*F*_c_^2^)/3
*wR*(*F*^2^) = 0.134	(Δ/σ)_max_ < 0.001
*S* = 1.17	Δρ_max_ = 0.41 e Å^−^^3^
4012 reflections	Δρ_min_ = −0.17 e Å^−^^3^
223 parameters	Absolute structure: Flack *x* determined using 1548 quotients [(*I*^+^)-(*I*^-^)]/[(*I*^+^)+(*I*^-^)] (Parsons *et al.*, 2013)
2 restraints	Absolute structure parameter: 0.01 (2)
Primary atom site location: structure-invariant direct methods	

### Special details

**Table:**

Geometry. All e.s.d.'s (except the e.s.d. in the dihedral angle between two l.s. planes) are estimated using the full covariance matrix. The cell e.s.d.'s are taken into account individually in the estimation of e.s.d.'s in distances, angles and torsion angles; correlations between e.s.d.'s in cell parameters are only used when they are defined by crystal symmetry. An approximate (isotropic) treatment of cell e.s.d.'s is used for estimating e.s.d.'s involving l.s. planes.
Refinement. Refinement of *F*^2^ against ALL reflections. The weighted *R*-factor *wR* and goodness of fit *S* are based on *F*^2^, conventional *R*-factors *R* are based on *F*, with *F* set to zero for negative *F*^2^. The threshold expression of *F*^2^ > σ(*F*^2^) is used only for calculating *R*-factors(gt) *etc*. and is not relevant to the choice of reflections for refinement. *R*-factors based on *F*^2^ are statistically about twice as large as those based on *F*, and *R*- factors based on ALL data will be even larger.

### Fractional atomic coordinates and isotropic or equivalent isotropic displacement parameters (Å^2^)

**Table:**

	*x*	*y*	*z*	*U*_iso_*/*U*_eq_	Occ. (<1)
C1	0.6776 (4)	0.5542 (4)	0.2160 (4)	0.0447 (8)	
C2	0.5707 (5)	0.6341 (4)	0.1270 (4)	0.0443 (8)	
C3	0.6859 (4)	0.3333 (4)	0.3536 (4)	0.0424 (7)	
C4	0.1894 (4)	1.0044 (4)	0.1824 (4)	0.0456 (8)	
C6	0.3256 (4)	0.7652 (4)	0.1360 (4)	0.0420 (7)	
C7	0.7162 (5)	0.1437 (5)	0.5065 (4)	0.0505 (9)	
H7	0.7053	0.1059	0.5854	0.061*	
C8	0.7473 (4)	0.2551 (4)	0.2713 (3)	0.0444 (8)	
H8	0.7567	0.2925	0.1916	0.053*	
C9	0.7798 (4)	0.0673 (4)	0.4242 (4)	0.0449 (8)	
C11	0.0916 (5)	0.9011 (5)	0.1102 (5)	0.0544 (10)	
H11	−0.0196	0.9119	0.0769	0.065*	
C12	0.1581 (4)	0.7821 (4)	0.0872 (4)	0.0507 (9)	
H12	0.0916	0.7125	0.0391	0.061*	
C13	0.6700 (5)	0.2740 (4)	0.4710 (4)	0.0494 (9)	
H13	0.6271	0.3242	0.5261	0.059*	
C14	0.7946 (5)	0.1233 (4)	0.3056 (4)	0.0463 (8)	
H14	0.8360	0.0725	0.2499	0.056*	
C15	0.6357 (5)	0.4749 (4)	0.3229 (4)	0.0535 (9)	
O4A	0.516 (5)	0.509 (5)	0.347 (7)	0.088 (5)	0.20 (6)
O4B	0.569 (3)	0.5385 (17)	0.390 (2)	0.088 (5)	0.80 (6)
C16	0.3938 (5)	0.6362 (4)	0.1112 (5)	0.0496 (9)	
C18	0.4208 (4)	0.8699 (4)	0.2085 (4)	0.0463 (8)	
H18	0.5320	0.8598	0.2417	0.056*	
C20	0.9065 (6)	−0.1371 (5)	0.3997 (6)	0.0724 (14)	
H20A	0.8372	−0.1526	0.3055	0.109*	
H20B	0.9380	−0.2221	0.4460	0.109*	
H20C	1.0004	−0.0888	0.4003	0.109*	
C21	0.3548 (4)	0.9895 (4)	0.2329 (4)	0.0456 (8)	
H21	0.4208	1.0587	0.2826	0.055*	
C22	0.2043 (6)	1.2268 (6)	0.2784 (6)	0.0742 (14)	
H22A	0.2795	1.2571	0.2367	0.111*	
H22B	0.2617	1.1972	0.3715	0.111*	
H22C	0.1337	1.2996	0.2800	0.111*	
O1	0.1126 (4)	1.1181 (3)	0.2000 (4)	0.0633 (8)	
O2	0.8234 (4)	−0.0603 (3)	0.4692 (3)	0.0620 (8)	
O3	0.3164 (4)	0.5356 (4)	0.0744 (6)	0.0929 (14)	
Cl1	0.62060 (13)	0.74187 (12)	0.01732 (11)	0.0625 (3)	
Cl2	0.87618 (12)	0.54992 (12)	0.22528 (13)	0.0651 (3)	

### Atomic displacement parameters (Å^2^)

**Table:**

	*U*^11^	*U*^22^	*U*^33^	*U*^12^	*U*^13^	*U*^23^
C1	0.0420 (17)	0.0381 (17)	0.0560 (19)	0.0057 (16)	0.0198 (15)	−0.0067 (16)
C2	0.0487 (19)	0.0387 (18)	0.0471 (19)	−0.0001 (15)	0.0186 (16)	−0.0032 (14)
C3	0.0382 (16)	0.0429 (19)	0.0446 (17)	0.0046 (15)	0.0126 (14)	−0.0020 (14)
C4	0.0460 (18)	0.0446 (19)	0.048 (2)	0.0078 (15)	0.0193 (16)	0.0042 (15)
C6	0.0404 (17)	0.0397 (19)	0.0452 (17)	0.0031 (15)	0.0141 (13)	0.0032 (14)
C7	0.052 (2)	0.053 (2)	0.049 (2)	−0.0062 (18)	0.0214 (17)	0.0025 (17)
C8	0.0446 (18)	0.048 (2)	0.0401 (16)	0.0033 (17)	0.0146 (14)	−0.0021 (15)
C9	0.0390 (16)	0.0403 (19)	0.0507 (19)	−0.0057 (15)	0.0098 (15)	−0.0021 (15)
C11	0.0343 (18)	0.061 (3)	0.064 (2)	0.0039 (17)	0.0122 (17)	−0.0033 (19)
C12	0.0400 (18)	0.050 (2)	0.058 (2)	−0.0061 (16)	0.0123 (16)	−0.0080 (17)
C13	0.050 (2)	0.052 (2)	0.052 (2)	0.0006 (17)	0.0252 (16)	−0.0062 (17)
C14	0.0454 (19)	0.0433 (19)	0.0496 (19)	0.0016 (16)	0.0161 (16)	−0.0090 (16)
C15	0.060 (2)	0.049 (2)	0.056 (2)	0.0144 (19)	0.0261 (19)	0.0014 (18)
O4A	0.131 (10)	0.066 (5)	0.099 (8)	0.046 (6)	0.079 (8)	0.018 (5)
O4B	0.131 (10)	0.066 (5)	0.099 (8)	0.046 (6)	0.079 (8)	0.018 (5)
C16	0.0439 (19)	0.0402 (19)	0.061 (2)	0.0017 (16)	0.0140 (17)	−0.0052 (17)
C18	0.0321 (16)	0.047 (2)	0.055 (2)	0.0029 (15)	0.0091 (15)	0.0002 (16)
C20	0.073 (3)	0.046 (2)	0.086 (3)	0.019 (2)	0.013 (2)	−0.001 (2)
C21	0.0411 (18)	0.0391 (18)	0.053 (2)	−0.0010 (15)	0.0124 (16)	−0.0070 (15)
C22	0.074 (3)	0.060 (3)	0.089 (3)	0.014 (3)	0.029 (3)	−0.018 (3)
O1	0.0521 (16)	0.0538 (19)	0.083 (2)	0.0161 (14)	0.0219 (15)	−0.0103 (16)
O2	0.072 (2)	0.0416 (16)	0.0703 (19)	0.0056 (14)	0.0220 (15)	0.0056 (13)
O3	0.0590 (18)	0.050 (2)	0.166 (4)	−0.0067 (17)	0.035 (2)	−0.032 (2)
Cl1	0.0694 (6)	0.0620 (6)	0.0619 (6)	0.0065 (6)	0.0302 (5)	0.0126 (5)
Cl2	0.0461 (5)	0.0549 (6)	0.0968 (8)	0.0105 (5)	0.0284 (5)	0.0012 (6)

### Geometric parameters (Å, º)

**Table:**

C1—C2	1.326 (5)	C9—O2	1.356 (5)
C1—C15	1.502 (6)	C11—H11	0.9300
C1—Cl2	1.732 (3)	C11—C12	1.377 (6)
C2—C16	1.521 (5)	C12—H12	0.9300
C2—Cl1	1.722 (4)	C13—H13	0.9300
C3—C8	1.393 (5)	C14—H14	0.9300
C3—C13	1.395 (6)	C15—O4A	1.219 (13)
C3—C15	1.473 (6)	C15—O4B	1.229 (8)
C4—C11	1.379 (6)	C16—O3	1.195 (5)
C4—C21	1.383 (5)	C18—H18	0.9300
C4—O1	1.362 (5)	C18—C21	1.384 (6)
C6—C12	1.402 (5)	C20—H20A	0.9600
C6—C16	1.476 (5)	C20—H20B	0.9600
C6—C18	1.380 (5)	C20—H20C	0.9600
C7—H7	0.9300	C20—O2	1.417 (6)
C7—C9	1.395 (6)	C21—H21	0.9300
C7—C13	1.367 (6)	C22—H22A	0.9600
C8—H8	0.9300	C22—H22B	0.9600
C8—C14	1.380 (6)	C22—H22C	0.9600
C9—C14	1.389 (6)	C22—O1	1.419 (6)
			
C2—C1—C15	121.2 (3)	C7—C13—H13	119.4
C2—C1—Cl2	121.4 (3)	C8—C14—C9	119.3 (3)
C15—C1—Cl2	117.1 (3)	C8—C14—H14	120.4
C1—C2—C16	122.9 (4)	C9—C14—H14	120.4
C1—C2—Cl1	122.5 (3)	C3—C15—C1	121.4 (3)
C16—C2—Cl1	114.6 (3)	O4A—C15—C1	117 (2)
C8—C3—C13	118.3 (3)	O4A—C15—C3	116 (2)
C8—C3—C15	123.6 (4)	O4B—C15—C1	115.8 (6)
C13—C3—C15	118.2 (3)	O4B—C15—C3	122.5 (6)
C11—C4—C21	120.5 (4)	C6—C16—C2	117.7 (3)
O1—C4—C11	115.9 (3)	O3—C16—C2	118.7 (4)
O1—C4—C21	123.6 (4)	O3—C16—C6	123.6 (4)
C12—C6—C16	119.3 (3)	C6—C18—H18	119.2
C18—C6—C12	118.4 (3)	C6—C18—C21	121.6 (3)
C18—C6—C16	122.3 (3)	C21—C18—H18	119.2
C9—C7—H7	120.0	H20A—C20—H20B	109.5
C13—C7—H7	120.0	H20A—C20—H20C	109.5
C13—C7—C9	119.9 (4)	H20B—C20—H20C	109.5
C3—C8—H8	119.3	O2—C20—H20A	109.5
C14—C8—C3	121.3 (4)	O2—C20—H20B	109.5
C14—C8—H8	119.3	O2—C20—H20C	109.5
C14—C9—C7	120.0 (4)	C4—C21—C18	119.0 (3)
O2—C9—C7	115.3 (3)	C4—C21—H21	120.5
O2—C9—C14	124.7 (4)	C18—C21—H21	120.5
C4—C11—H11	119.9	H22A—C22—H22B	109.5
C12—C11—C4	120.1 (3)	H22A—C22—H22C	109.5
C12—C11—H11	119.9	H22B—C22—H22C	109.5
C6—C12—H12	119.8	O1—C22—H22A	109.5
C11—C12—C6	120.4 (4)	O1—C22—H22B	109.5
C11—C12—H12	119.8	O1—C22—H22C	109.5
C3—C13—H13	119.4	C4—O1—C22	119.3 (3)
C7—C13—C3	121.2 (4)	C9—O2—C20	117.7 (4)
			
C1—C2—C16—C6	121.1 (4)	C13—C7—C9—C14	−0.6 (6)
C1—C2—C16—O3	−61.8 (6)	C13—C7—C9—O2	179.4 (4)
C2—C1—C15—C3	137.3 (4)	C14—C9—O2—C20	7.5 (6)
C2—C1—C15—O4A	−17 (4)	C15—C1—C2—C16	−9.4 (6)
C2—C1—C15—O4B	−48.1 (18)	C15—C1—C2—Cl1	171.9 (3)
C3—C8—C14—C9	0.3 (6)	C15—C3—C8—C14	179.1 (4)
C4—C11—C12—C6	−0.5 (7)	C15—C3—C13—C7	−179.1 (4)
C6—C18—C21—C4	−0.5 (6)	C16—C6—C12—C11	179.4 (4)
C7—C9—C14—C8	0.6 (6)	C16—C6—C18—C21	−178.9 (4)
C7—C9—O2—C20	−172.5 (4)	C18—C6—C12—C11	0.7 (6)
C8—C3—C13—C7	1.3 (6)	C18—C6—C16—C2	−18.6 (6)
C8—C3—C15—C1	−12.4 (6)	C18—C6—C16—O3	164.4 (5)
C8—C3—C15—O4A	142 (4)	C21—C4—C11—C12	−0.3 (7)
C8—C3—C15—O4B	173.5 (19)	C21—C4—O1—C22	−2.4 (7)
C9—C7—C13—C3	−0.4 (6)	O1—C4—C11—C12	−180.0 (4)
C11—C4—C21—C18	0.8 (6)	O1—C4—C21—C18	−179.6 (4)
C11—C4—O1—C22	177.3 (4)	O2—C9—C14—C8	−179.4 (3)
C12—C6—C16—C2	162.7 (4)	Cl1—C2—C16—C6	−60.1 (4)
C12—C6—C16—O3	−14.2 (7)	Cl1—C2—C16—O3	117.0 (5)
C12—C6—C18—C21	−0.2 (6)	Cl2—C1—C2—C16	177.2 (3)
C13—C3—C8—C14	−1.3 (5)	Cl2—C1—C2—Cl1	−1.5 (5)
C13—C3—C15—C1	168.0 (4)	Cl2—C1—C15—C3	−49.0 (5)
C13—C3—C15—O4A	−38 (4)	Cl2—C1—C15—O4A	157 (4)
C13—C3—C15—O4B	−6.1 (19)	Cl2—C1—C15—O4B	125.5 (17)

### Hydrogen-bond geometry (Å, º)

**Table:**

*D*—H···*A*	*D*—H	H···*A*	*D*···*A*	*D*—H···*A*
C7—H7···O4*A*^i^	0.93	2.49	3.23 (3)	137
C7—H7···O4*B*^i^	0.93	2.62	3.245 (9)	125
C12—H12···O1^ii^	0.93	2.66	3.488 (5)	149
C14—H14···O1^iii^	0.93	2.72	3.363 (5)	127

Symmetry codes: (i) −*x*+1, *y*−1/2, −*z*+1; (ii) −*x*, *y*−1/2, −*z*; (iii) *x*+1, *y*−1, *z*.
