# Supplementary figures and images for: Crystal structure of (Z)-2,3-di­chloro-1,4-bis­(4-meth­oxy­phen­yl)but-2-ene-1,4-dione
Source: Acta Crystallogr Sect E Struct Rep Online. 2014 Aug 23;70(Pt 9):o1049–50. doi: 10.1107/S1600536814018790 (PMC4186131; doi:10.1107/S1600536814018790)

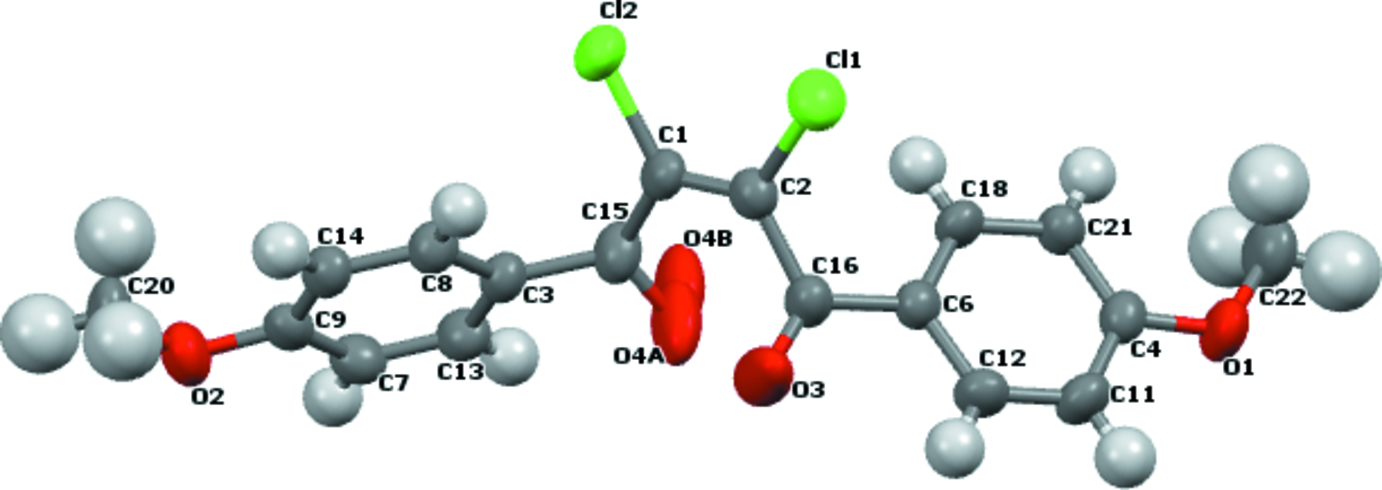

Supplement: Supplementary file 4 [file e-70-o1049-fig1.tif]

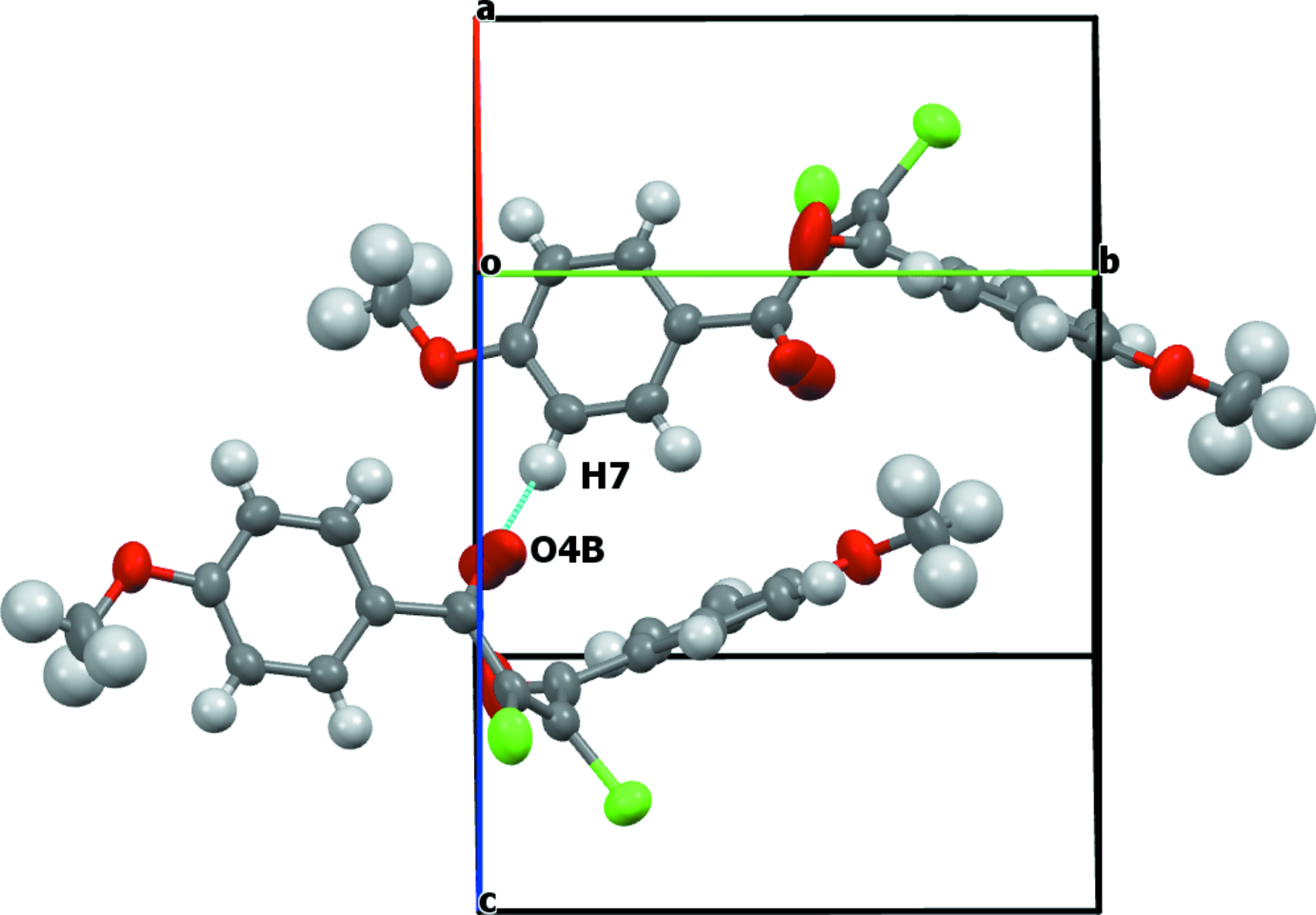

Supplement: Supplementary file 5 [file e-70-o1049-fig2.tif]

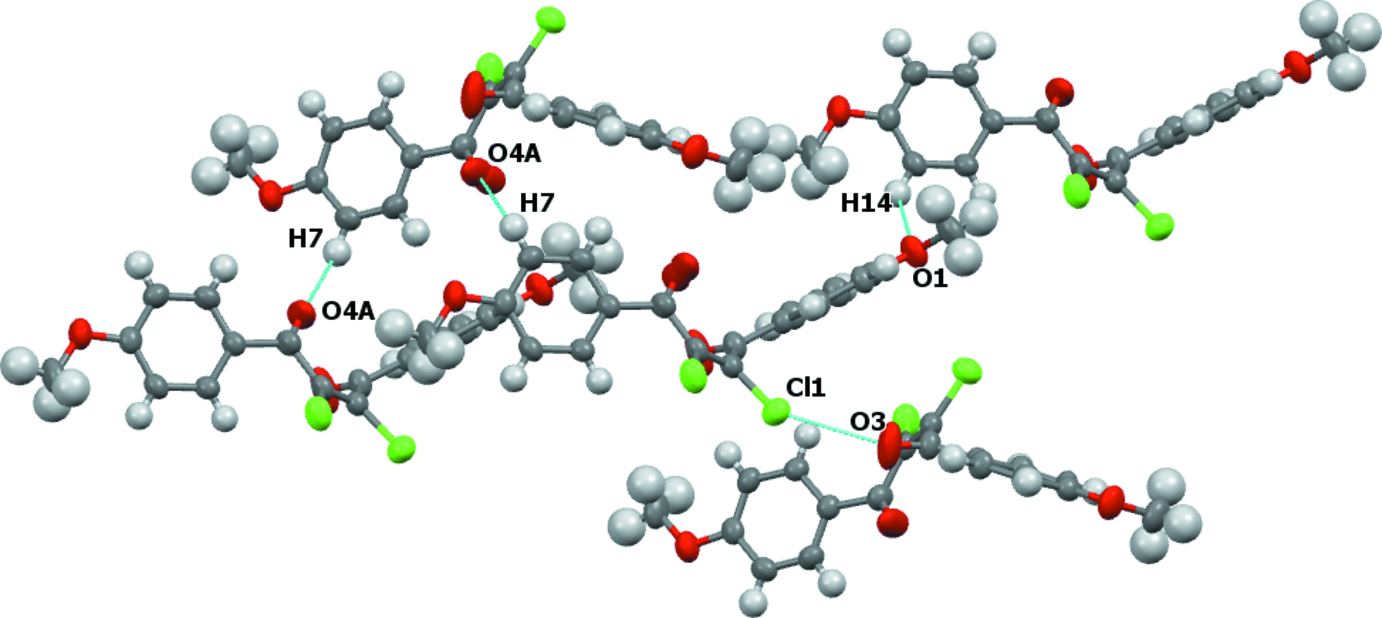

Supplement: Supplementary file 6 [file e-70-o1049-fig3.tif]
